# Kyphectomy in Myelomeningocele for Severe Early-Onset Kyphosis Using Distal Intravertebral Fixation and Thoracic Growing Rods

**DOI:** 10.5435/JAAOSGlobal-D-19-00006

**Published:** 2019-09-23

**Authors:** Khalid Saud Alshaalan, Jason J. Howard, Ahmed Khaled Alshangiti, Yasser I. Alkhalife, Sami Aleissa, Samir Omar Al Sayegh

**Affiliations:** From the College of Medicine, King Saud bin Abdulaziz University for Health Sciences, Riyadh, Saudi Arabia (Dr. Alshaalan, Dr. Alshangiti, Dr. Aleissa, and Dr. Al Sayegh); Department of Orthopedic Surgery, Nemours/Alfred I. duPont Hospital for Children, Wilmington, Deleware (Dr. Howard); and the King Abdullah Specialized Children Hospital (KASCH), King Abdulaziz Medical City (KAMC), Riyadh, Saudi Arabia (Dr. Alshaalan, Dr. Alshangiti, Dr. Alkhalife, Dr. Aleissa, and Dr. Al Sayegh).

## Abstract

Most kyphectomy techniques require distal dissection of the bifid posterior spinal elements for implants placement in the thoracolumbar/pelvic regions, traversing the scarred tissue associated with previous MMC closure, thereby theoretically increasing the risk of wound complications. The *Halifax kyphectomy* technique avoids the MMC scar but does not reliably facilitate thoracic growth for early-onset kyphosis. This study aims to report the technique and outcomes of a combined Halifax kyphectomy (resection of the apical vertebrae with distal anterior multilevel vertebral body fixation) and thoracic growing rod construct used to treat early-onset symptomatic gibbus in a patient with myelomeningocele (MMC).

**Methods:**

A 3-year-old girl with a thoracic MMC presented with symptomatic gibbus requiring surgical intervention. Correction by the Halifax kyphectomy technique combined with spine-based growing rods was performed.

**Results:**

After the correction, the skin was closed primarily without the need for any flap for coverage. No wound complications or infection occurred post-operatively. The intraoperative blood loss was 200 mL, and the surgical time was 419 minutes. No pulmonary complications occurred postoperatively. At the final follow-up at 3 years 11 months postoperatively, the child had no recurrence of the deformity.

**Conclusions:**

The combination of distal anterior multilevel vertebral body fixation with spine-based thoracic growing rods can successfully achieve kyphosis correction in MMC, with the potential to reduce complication rates and facilitate thoracic growth. Further investigation is necessary to prove whether the outcomes and the complication rates are superior to other established techniques.

The incidence of severe kyphosis associated with myelomeningocele (MMC) is reported to be approximately 12% to 20% and is related to the neurosegmental level, with higher level lesions being at increased risk.^1–3^ In most cases, the associated gibbus requires only seating modifications and supportive care. However, functional concerns may complicate its natural history, among which skin breakdown over the apex of the deformity and concomitant infection are important.^4^ These and other functional issues, including impaired sitting balance, poor body image, discomfort, truncal growth retardation, and thoracic insufficiency syndrome (TIS),^5^ are relative indications for surgical management.

The most common surgical intervention involves resection of the vertebrae comprising the gibbus and fusion of the proximal and distal segments (ie, kyphectomy). In 1968, Sharrard^6^ first described the successful management of a kyphosis case associated with MMC. Since then, many other techniques have been developed.^1,3,7–9^ Despite these developments, the complication rates remain extremely high, especially the risk of deep wound infection.^8,9,10,11,12,13,14^

Most kyphectomy techniques require distal dissection of the bifid posterior spinal elements for the placement of implants in the thoracolumbar/pelvic regions, traversing the scarred tissue associated with the previous MMC closure, and theoretically increasing the risk of postoperative infection. To avoid this compromised area, similar techniques have been reported by centers in Canada (the so-called *Halifax kyphectomy*)^15^ and Australia,^4^ which avoid the MMC closure scar. These techniques require dissection just distal to the apex of the gibbus; in the aforementioned case series, favorable complication rates over those of other techniques were reported.^4,15^

As symptomatic gibbus in MMC often has an early onset, procedures that maintain thoracic growth must be considered to avoid TIS. To our knowledge, a technique that combines the benefits of the Halifax kyphectomy and spine-based growing rods has not been previously described. We report a case wherein this combined technique was used to treat a thoracolumbar gibbus. The Institutional Review Board of King Abdullah International Medical Research Center approved this study, and consent for publication was obtained from the patient's family.

## Case Report

**Figure 1:**
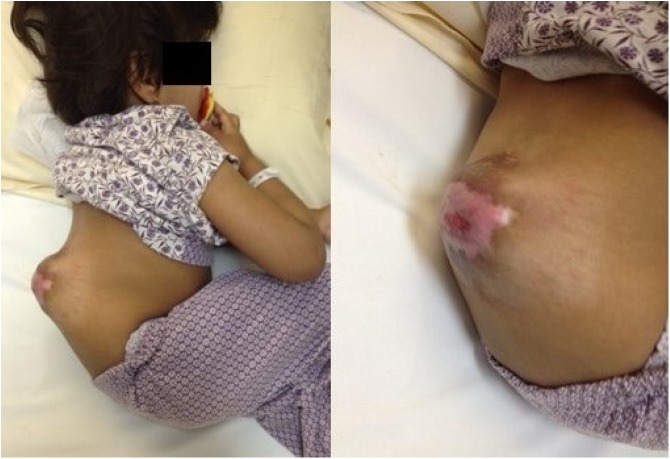
Clinical images of the acute gibbus in a 3-year-old girl with myelomeningocele. Ulceration is seen at the apex of the gibbus with visible previous scarring.

**Figure 2:**
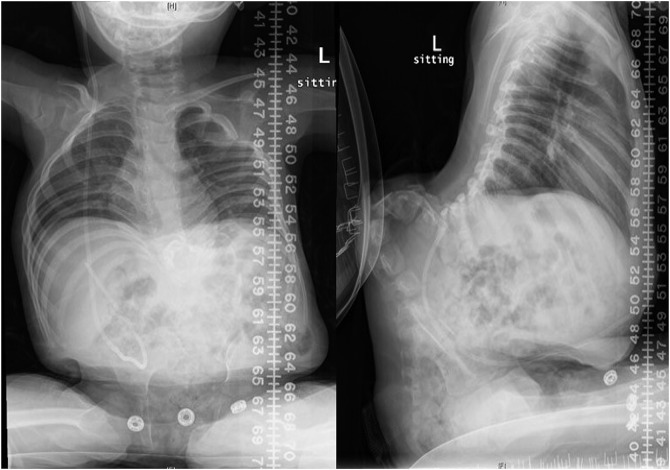
AP and lateral radiographs of the thoracolumbar kyphotic deformity. The posterior spinal elements are deficient at the periapical region. The apex of the gibbus is at L2.

A 3-year-old girl with MMC at the thoracic level, progressive thoracolumbar gibbus deformity, and pressure ulceration presented to our clinic. The MMC closure occurred shortly after birth. A clinical examination revealed skin ulceration with previous scarring at the apex of gibbus deformity (Figure 1). Other related history and physical examination findings were normal. Radiographs demonstrated a severe kyphotic deformity just distal to the thoracolumbar junction (Figure 2).

As recurrent skin ulceration was noted over the gibbus, surgical correction of the kyphosis using a Halifax kyphectomy technique and growing rod insertion was performed. The patient was placed in the prone position on bolsters. Due to the risk of aortic stretch (and even rupture) with large kyphectomy corrections, pulse oximeters were placed at each lower limb to detect any lower limb vascular compromise during the correction maneuver.^16^ Preincision antibiotics including a first-generation cephalosporin and gentamicin were given. A 4-cm midline posterior spinal incision was made at the upper thoracic spine under fluoroscopic guidance, followed by subperiosteal dissection of the posterior elements at the T2/T3 vertebrae and subsequent placement of pedicle screws bilaterally at these levels. A 4.5-mm rod posterior spinal system was used. After this, an elliptical incision was performed to excise the ulcer over the gibbus. The neural placode was dysplastic at the apex of kyphosis and was subsequently mobilized and preserved. We then identified the junction of the normal thoracic spine (T9) and the start of the bifid distal laminae (T10). Pedicle screws were inserted bilaterally at T9/T10 under fluoroscopic guidance. After inserting all pedicle screws, the vertebral resection was performed.

**Figure 3:**
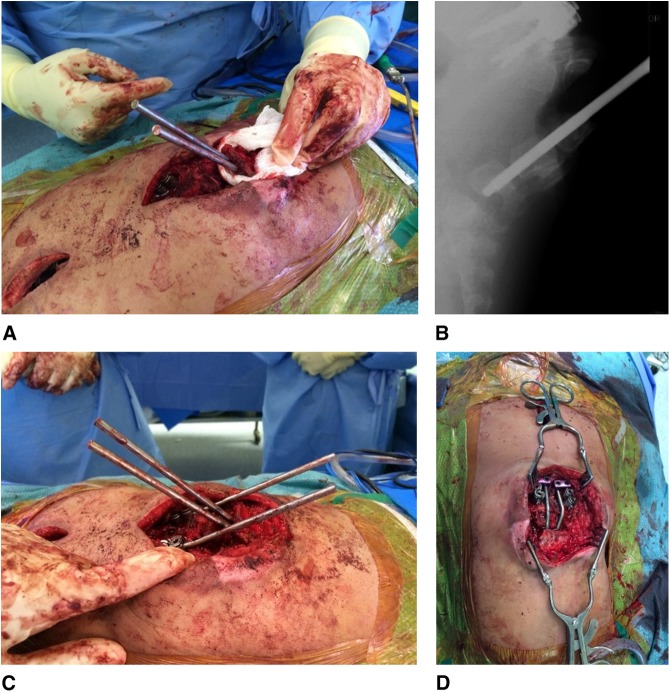
**A**, Titanium rods were inserted through the superior end plate of the L3 vertebral body through to the inferior end plate of L5. **B**, Lateral intraoperative radiograph showing insertion of spinal rods through the vertebral bodies of L3, L4, and L5. **C**, The distal rod ends that formed the proximal growing rod construct were left long at this point to allow for connection to the distal rod construct. The proximal and distal rod constructs were then brought together in a drawbridge maneuver to correct the kyphosis and connect the proximal and distal instrumentation. The proximal upper thoracic incision for insertion of the T2/T3 pedicle screws is visible at the left-hand side of the image. **D**, The connection between the distal rod construct—bent accordingly to facilitate connection to the domino connectors attached to the proximal growing rod construct. The pedicle screws at T9/T10 are visible at the proximal extent of the incision.

The apical vertebrae were carefully exposed via extraperiosteal dissection circumferentially around the vertebrae. Resection of the kyphosis was performed with complete removal of L2 and L1 and partial removal of T12. Two adjacent 4-mm drill holes were made through the middle of the L3, L4, and L5 vertebral bodies, and a 4.5-mm straight titanium rod was inserted into each hole (Figure 3).

**Figure 4:**
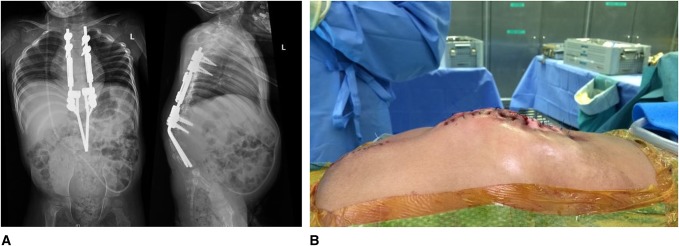
**A**, Immediate postoperative AP and lateral radiographs showing the correction of the kyphosis. Traditional growing rod construct is noted in the thoracic spine, connected by domino correctors to the distal construct. The convergence of the distal rods helps resist implant pullout. **B**, Intraoperative image immediately after wound closure. The skin closed easily without flaps or preoperative skin expanders.

Using standard technique,^17^ the 4.5-mm growing rods with 40-mm-long tandem connectors were tunneled in a submuscular, extraperiosteal fashion and were connected to the pedicle screw constructs at the upper and lower thoracic spine. The distal rod ends were left long at this point to allow for connection to the distal rod construct. The proximal and distal rod constructs were then brought together in a *drawbridge* maneuver and were used as lever arms to reduce the osteotomy and correct the kyphotic deformity (Figure 3). A domino connector was then used to attach the growing rod construct to the distal rod construct (Figure 3). This provided an excellent correction of the kyphosis, confirmed with intraoperative radiograph. After correction, primary skin closure was performed with no flap required for coverage (Figure 4). The intraoperative blood loss was approximately 200 mL, and the surgical time was 419 minutes.

**Figure 5:**
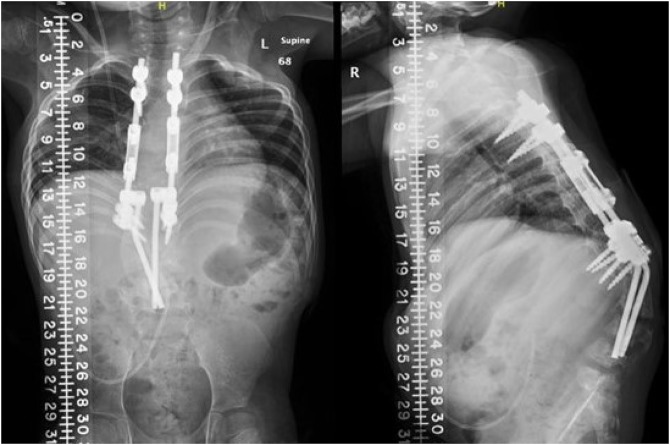
Subsequent AP and lateral radiographs after growing rod expansions at 3 years postoperatively. Proximal migration of the distal construct is noted as the lumbar spine *grows off* the rods.

**Figure 6:**
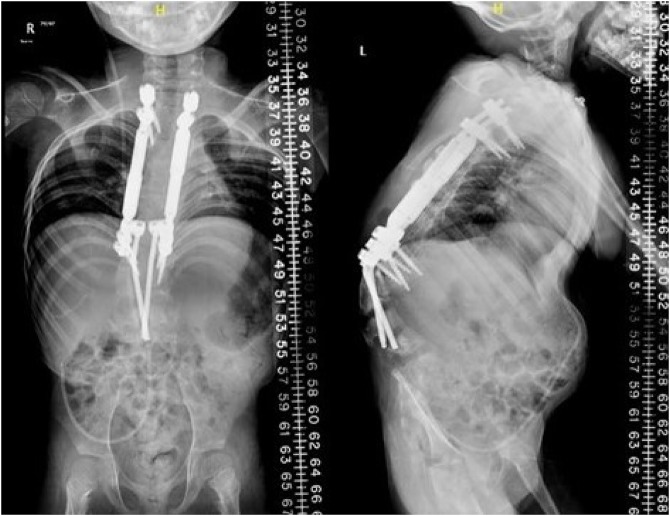
Subsequent AP and lateral radiographs after fixation with magnetically controlled growing rod expansions. The rod has cut-out at the superior aspect of the L5 body. This was due to the lumbar spine *growing off* the rods distally, which is one of the expected outcomes (and benefits) of this technique.

The patient tolerated the procedure well and had no wound complications or infection postoperatively. Growing rod expansion was performed once every 6 months, and four expansions were completed before the rods were outgrown, necessitating rod exchange (Figure 5). No signs of infection, loosening, or pullout at that time was noticed. At 6 years of age, the conventional growing rods were revised to a magnetically controlled growing rod system (Figure 6). At the time of revision surgery, a minor cut-out was observed at the superior aspect of the L5 body. This was due to the lumbar spine *growing off* the rods distally, which is one of the expected outcomes (and benefits) of this technique.

At the final follow-up at 3 years 11 months postoperatively, the patient had no pain, no seating intolerance, and no recurrence of the ulcer. The kyphosis was reduced from 155° preoperatively to 85° postoperatively (45% correction), with no progression of the deformity at the final follow-up. No significant pulmonary infections or other respiratory compromise occurred during the follow-up period.

## Discussion

Kyphectomy has become one of the more common indications for spinal correction in MMC given recent evidence suggesting that scoliosis correction in these patients does not improve quality of life and has a high risk of complications.^18^ Various techniques for fixation and correction following kyphectomy have been reported. In early reports, short posterior fusion procedures using staples, surgical wires, and screws resulted in significant correction loss in most cases.^3,10,11^ In the modern era, most surgeons perform long posterior fusion for kyphectomy correction.^6,19^ The most common postoperative concerns associated with kyphectomy include wound-related complications, including dehiscence and deep wound infection which have been reported to be as high as 89% in one series.^13^ In larger series, infection rates following the more *traditional* method of kyphectomy range between 4.5% and 12.5%, with wound-related complications being even more prevalent.^9,20,21,22,23^ Significant rates of pseudarthrosis and implant failure have also been reported.^8,9,19,20^

One of the advantages of the Halifax kyphectomy technique is the avoidance of the MMC scar. We believe this avoidance decreases the risk of wound-related complications and postoperative infection by operating through a tissue that is relatively healthy. Accordingly, our patient did not experience any wound dehiscence or infection. In addition, no deep wound infections were reported for either the Halifax or Australian series, both of which used the kyphectomy technique used in this report.^18,24^

Given the young age at which many of these patients require kyphectomy, correction techniques that preserve spinal growth and prevent TIS are desired. The technique described herein addresses issues related to both wound complications and the preservation of spinal growth by combining thoracic spine-based growing rods with anterior intravertebral rod fixation of the lumbar vertebrae without fusion. This is in contrast to more commonly performed techniques that involve posterior instrumentation and fusion from T2 to the pelvis, which could at least lead to partial cessation of spinal growth. Except for the limited fusion at the osteotomy site, the combined procedure of Halifax kyphectomy and thoracic growing rods insertion allows for continued growth of the entire spine (Figure 6).

When the initial procedure was performed, we did not have access to magnetically controlled growing rods. Therefore, multiple open growing rod lengthening procedures were performed for the first few years following the index procedure, in a standard fashion via a small midline incision made over the tandem connectors.^17^ At the time of revision, these implants were replaced with magnetically controlled rods, eliminating the need for surgery for future lengthening procedures. This implant choice, coupled with the lack of distal dissection into the region covered by the often-precarious skin associated with the MMC closure, should help further reduce the risk of infection, a devastating complication all too common after spinal surgery in MMC.^25^ In our case, no infection was identified; however, whether this was a result of the technical choices unique to this procedure is not known.

In this case, due to the child's size, we chose 4.5-mm titanium rods for distal anterior vertebral fixation. In practice, however, it is best to choose the largest diameter rod possible (preferably 6.0 mm or above) which is made of the stiffest material (eg, cobalt-chromium) to avoid rod fracture in this nonfusion construct.^4,18^

In this case, the intraoperative blood loss was 200 mL and the surgical time was 419 minutes. Comstock et al assessed the blood loss associated with the Halifax kyphectomy and found it to be 765 mL, substantially lower than in other kyphectomy reports.^18^ The decreased blood loss in our case and in Comstock's series was likely due to the reduced distal exposure required as compared with other techniques.

A potential disadvantage of this technique over traditional techniques that use segmental posterior instrumentation is the lack of the ability to restore a more normal lumbar lordosis in exchange for a straight distal lumbosacral segment. That said, the goal of kyphectomy correction is to prevent recurrent skin ulceration at the gibbus, which was reliably achieved in our patient and in other series using the Halifax kyphectomy technique.^24^ Another potential disadvantage is the lack of iliac fixation which may decrease the efficacy of pelvic obliquity correction when needed. Despite this theoretical concern, we feel that the stable anterior multilevel intravertebral fixation achieved with the Halifax kyphectomy technique allows for a cantilever to reduce both sagittal and coronal plane deformity in the lumbosacral spine, particularly when the distal extent of fixation extends to S1 or below, as is technically feasible.^15,24^

According to this report, the combination of distal anterior multilevel vertebral body fixation with spine-based thoracic growing rods can successfully achieve kyphosis correction in MMC and it has the potential to reduce complication rates by minimizing blood loss, wound dehiscence, and deep wound infection while facilitating thoracic growth. Further investigation is necessary to prove whether the outcomes and the complication rates are superior to other established techniques.
